# Merging the Social Influence Theory and the Goal-Framing Theory to Understand Consumers’ Green Purchasing Behavior: Does the Level of Sensitivity to Climate Change Really Matter?

**DOI:** 10.3389/fpsyg.2021.766754

**Published:** 2021-11-01

**Authors:** Xianchuan Yang, Yafen Tseng, Beyfen Lee

**Affiliations:** ^1^School of Business, Wuxi Vocational Institute of Commerce, Wuxi, China; ^2^Social Science Applied Research and Synergetic Innovation Bases in Jiangsu Province, Wuxi, China; ^3^Digital Design and Information Management, Chung Hwa University of Medical Technology, Tainan, Taiwan; ^4^Department of Hospitality Management, Chung Hwa University of Medical Technology, Tainan, Taiwan

**Keywords:** social influence, goal-framing theory, green purchasing behavior, activation ability, sensitivity to climate change

## Abstract

This study explored the formation of consumers’ green purchasing behavior (GPB) and investigated the moderating effect of sensitivity to climate change (SCC) to address this current knowledge gap. An integrated model merging the Social Influence Theory and the Goal-framing Theory was developed with the Stimulus-Organism-Response (S-O-R) paradigm. An empirical study was conducted, surveying 583 respondents and analyzing the questionnaire results using structural equation modeling. The results show that media, family, and peer influence (PEI) can effectively activate the consumers’ goal frames. Hedonic and normative goals had significant positive influences on GPB, while gain goals had no significant effect. SCC was found to significantly moderate social influence on GPB through the consumers’ goal frames. This research provided strong empirical support on understanding the relationship between social influence and GPB through three goal frames. In addition, the potential differences of the GPB formation process in two subgroups (high SCC and low SCC) are also investigated. The results of this study can help green practitioners develop more effective marketing strategies and incentives targeted to consumers with varying levels of environmental consciousness or sensitivity.

## Introduction

Given mounting environmental challenges, people have increasingly become more mindful of environmental concerns (Liu et al., 2016; Wang Y. et al., 2019) and the importance of prioritizing sustainable development (Hosta and Zabkar, 2021). Green consumption has become an important issue for governments, businesses, and consumers (Ge et al., 2020), as about 40% of environmental deterioration can be traced directly from residential non-green consumption (Moon et al., 2019). However, many still do not practice green purchasing behaviors (GPBs), especially among apathetic consumers. While most governments are dedicated to green market development, environmental governance efficiency in the green market is significantly mired by numerous barriers (Carrington et al., 2010; Tu et al., 2020). For green marketers, green consumption barriers are making green marketing strategies come to nothing (Chen et al., 2020). Dealing with barriers to GPB is crucial to achieving environmental consumption and long-term sustainable development (Yang and Zhang, 2020).

Facing environmental crises, various social forces (i.e., media, family, and peers) have given full attention to these crises. Accordingly, green consumerism has begun to spring up (Lee, 2011; Ivanova et al., 2019). Studies on green consumption increase rapidly from different theoretical perspectives and approaches (Liu et al., 2016), focusing on identifying drivers to green consumption (intention) behaviors (e.g., Chen, 2013; Wang Y. et al., 2019). Especially, it is becoming increasingly common to build theoretical framework on multiple theories such as the Stimulus-Organism-Response (S-O-R) model, Social Influence Theory and The Goal-framing Theory, or their extended (integrated) model, aiming to predict pro-environmental attitude or behavior (Kiatkawsin and Han, 2017; Su and Swanson, 2017). However, while the drivers derived from current research approaches can activate positive green attitude (motivation), they cannot effectively transform positive green attitude (motivation) into GPB, resulting in attitude (motivation)-behavior gap (Carrington et al., 2010; Claudy et al., 2013; Tang et al., 2020; Yang and Zhang, 2021). Therefore, there is a strong need to narrow the attitude (motivation)-behavior gap.

According to the S (Stimulus) – O (Organism) – R (Response) model (Mehrabian and Russell, 1974), GPB, a typical acquired behavior, is an effective response to the stimulus. External stimuli and example-setters could shape, reshape, and change individuals’ pro-environmental psychology and behaviors (Lee, 2011; Choi and Kandampully, 2019; Yang and Zhang, 2021). Some studies have been focused on influencing factors such as consumption value (Lin and Huang, 2012; Biswas and Roy, 2015; Wang Y. et al., 2019), consumption motivations (Ofek and Portnov, 2020), social norms (Kiatkawsin and Han, 2017; Ge et al., 2020), social culture (Jiang et al., 2020), social identity (Pinto et al., 2014), and fear appeals (Moon et al., 2019; Wang L. et al., 2019). However, many of these studies fail to explore and determine the formation mechanism of human acquired/complex behavior. Although those factors can, to some extent, predict GPB, they lack system and causal disorder. Most existing studies have only explored the influence mechanism of stimulus factors on individuals’ green consumption attitude or behavior based on scattered variables (Ivanova et al., 2019), while human learning and cognitive rules have been ignored. In addition, owing to the divorce of the public information campaign and interpersonal interaction learning, the predictive power of stimuli on complex acquired behaviors (e.g., GPB) has been weakened.

Moreover, Psychological traits refer to the psychological structure that can make people’s behavior and disposition exhibit persistence, stability, and consistency (Costa and McCrae, 1992; Wei et al., 2016), which suggests that the differences in mental characteristics may cause differentiated social behavior. For psychological traits and pro-environmental behavior, however, the relationship is vague and unsettled. One view posits that psychological traits, such as fear and neuroticism, negatively influence the formation process of pro-environmental behavior (Janis and Feshbach, 1953; Yang and Zhang, 2020). In contrast, another view suggests that individuals with deep fear are more sensitive to environmental problems (e.g., Suki and Suki, 2019; Wang L. et al., 2019). Their emotions are more receptive to adverse environmental consequences, thus promoting pro-environmental motivations and inducing actual behaviors (Wiseman and Bogner, 2003).

Similarly, as a psychological trait, varying levels of sensitivity to climate change (SCC) may considerably moderate the formation process of GPB. Several studies have investigated the moderated role of environmental literacy (e.g., environmental concern and environmental consciousness) in their theoretical models (Lin and Huang, 2012; Kautish et al., 2019). Kim and Hwang (2020) argued that the relative importance of explanatory variables in predicting behavior varies when individuals possess different levels of product knowledge. However, very little attention has been paid to the moderated role of SCC. Thus, this paper introduces SCC into an integrated model.

To address the current research gaps, this study proposes a research framework using the S-O-R model, which integrates and optimizes the relationships among the existing variables. The proposed framework views social influence (SI) as a multidimensional construct, which encompasses media exposure (ME), family influence (FAI), and peer influence (PEI; Lee, 2010; Ivanova et al., 2019). Lindenberg and Steg (2007) explored the relationship between goal frames and environmental behavior from the perspective of egoistic and altruistic appeals, but they ignored how the individual’s goal frames are activated by the stimuli. In this paper, the goal-framing theory is also introduced, using three-goal frames as mediators. The dual appeal to individuals was segmented into gain goal frames (GGFs), hedonic goal frames (HGFs), and normative goal frames (NGFs), thus exploring the influence mechanism of SI on GPB that simultaneously considers dual appeals. In addition, this study will investigate the potential moderating role of SCC on the research model, and an empirical study was conducted using a questionnaire survey with 583 respondents. The findings of this study will help explain the motivation-behavior gap and heterogeneity in the GPB formation process, providing theoretical and practical guidance for green consumption policies and strategies.

## Theoretical Framework and Hypothesis

### The Stimulus-Organism-Response Model Converging Two Theories

The S-O-R model, proposed by Mehrabian and Russell (1974), argues that there is a “mediated process” between the stimuli and response and describes the changes in the recipient’s psychological state after receiving external stimuli (Choi and Kandampully, 2019). The S-O-R model was one of the earliest frameworks used in exploring the impact of various stimuli on pro-environmental behavior (e.g., Su and Swanson, 2017; Choi and Kandampully, 2019). In this model, stimulus (S), which is based on context and object, is the sum of all external driving factors without a specific range. For the organism (O), aroused motivations are indicated by psychological activities, while the individual’s behavioral outcome (R) denotes GPB. Actually, most scholars agree that the existing theories are imperfect (Kim and Hwang, 2020), and a number of criticisms have been raised (Kiatkawsin and Han, 2017). For the Goal-framing Theory, this approach fails to reveal the formation process of goal frames. More importantly, previous studies have found that the integrated model exhibit stronger predictive power than the standalone theory (Kiatkawsin and Han, 2017). Accordingly, this paper proposes an S-O-R framework, incorporating the Social Influence Theory and the Goal-framing Theory, to better understand GPB formation.

Social influence (SI) refers to the social and psychological phenomenon in which the individual’s thoughts, attitudes, and behavior are changed by external social pressure (Kelman, 1974). Various models and frameworks have been proposed explaining and rationalizing how social influence develops and translates into actions. Kelman (1974) proposed that social influence has three social processes: compliance, identification, and internalization. In this study, ME, FAI, and PEI were investigated.

In Lindenberg and Steg (2007) proposed the Goal-framing Theory, which posits that three goal frames guide environmental behavior: GGFs, HGFs, and NGFs. GGF reflects the individual’s perceived maximization of utility and benefits (Lindenberg and Steg, 2007; Tang et al., 2020), while HGF manifests a person’s feelings in a specific situation, seeking pleasure, and excitement. NGF makes people more sensitive to what they think should be done and leads them to take appropriate actions, such as pro-environmental behaviors (Lindenberg and Steg, 2007). The Goal-framing Theory argues that people’s behaviors result from multiple motivations that jointly influence their thoughts, sensitivities, and actions in a given situation (Tang et al., 2020). This theory adopts an integrative framework that takes into account egoistic (GGF and HGF) and altruistic (NGF) appeals in explaining behavior (Tang et al., 2020). In our study, the GGF, HGF, and NGF act as the “mediated process,” namely organism.

**FIGURE 1:**
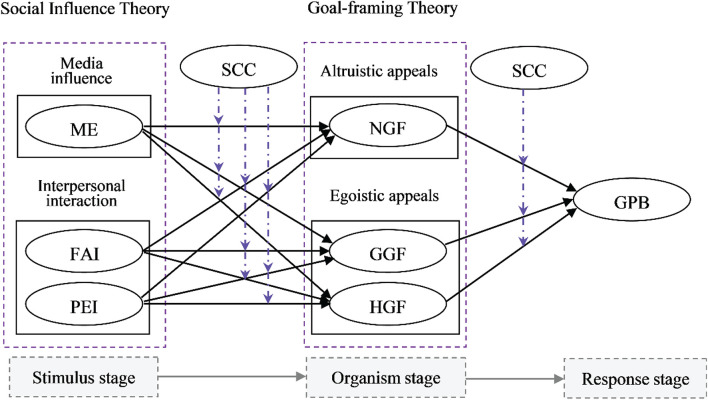
Theoretical framework.

Additionally, this study hypothesized that consumers’ levels of SCC may moderate the causal relationships among the variables. Figure 1 presents the theoretical framework.

### Hypothesis

#### Media Exposure and Goal Frames

Media is an organizer, guide, participant, and supervisor in environmental protection, and ME refers to the frequency of environmental issues in programs, news, and various advertisements that guide the audience’s environmental attitude and behavior (Lee, 2010, 2011; Tu et al., 2020; Yang and Zhang, 2020; Hosta and Zabkar, 2021). Previous studies have found that media can guide the public to pay attention to environmental issues by setting an environmental agenda (McCombs, 1997), which generates collectivistic (altruistic appeals) and individualistic (egoistic appeals) values in green consumption, and shapes positive green purchasing motivation (Lindenberg and Steg, 2007).

When the media provides environmental crisis information, consumers reappraise their perceived gains and losses, which modify green perceived value. To avoid or mitigate harm from environmental crises, buyers become more aware of green functional value, which breaks the stereotype of green products being unjustifiably high priced (Chen, 2013; Tu et al., 2020; Xu et al., 2020). ME can strengthen consumers’ perceived seriousness of environmental problems (Yang and Zhang, 2020) and make them pay more attention to incentives for green consumption (e.g., financial subsidies, old for new) (Tang et al., 2020). The generated appeal for added benefits (conditional value) from green consumption would then help accelerate the formation of GGF (Gonçalves et al., 2016).

Hedonic goal frame is an important part of consumption value. It differs from utilitarian value, which is task-oriented, as HGF reflects the pleasure and positive emotions experienced during shopping (Babin et al., 1994; Gonçalves et al., 2016; Xu et al., 2020). Given mounting environmental concerns, ME can gradually reshape a new lifestyle that advocates for green consumption, promotes identity for ecological citizenship, and increases hedonic motivations for conservation (Pinto et al., 2014; Xu et al., 2020). In addition, to avoid potential environmental harm, consumers may develop preference for green consumption and generate positive emotional motivation, thus avoiding negative experiences and various uncertainties (Lindenberg and Steg, 2007; Wang et al., 2017).

Media exposure can also effectively reshape green social norms (Lee, 2010) and modify social ethics. To gain rewards or avoid punishment, consumers are more likely to follow social expectations and moral standards, enforce ecological justice (Zhang et al., 2013), and bear more environmental responsibilities (Hosta and Zabkar, 2021). According to the Norm Activation Model (Schwartz and Howard, 1981), crisis information revealed in the media deepens the ascription of responsibility and awareness of the consequences for not implementing pro-environmental behaviors, thus generating motivation to conform to social norms. Pinto et al. (2014) stated that to obtain group identity, individuals would consciously take social norms as their own code of conduct to enhance the sense of belonging to the ecological citizen group. Based on these arguments, the following hypothesis is proposed:

H1: *ME positively influences consumers’ GGF (H1a), HGF (H1b), and NGF (H1c).*

#### Family Influence and Goal Frames

Family members are the first social forces to instill values and behavior into individuals (Lee, 2011). Family members instill egoistic and altruistic appeals simultaneously based on the bounded self-serving and bounded ethicality concepts (Yang and Zhang, 2020). They can actively share environmental knowledge with their relatives, increasing their environmental awareness, and persuading them to adopt healthier lifestyles (e.g., green lifestyle) to avoid environmental harm (Ivanova et al., 2019; Xu et al., 2020). Individuals may then actively seek green product information and continuously improve their green consumption skills. Haryanto (2014) argues that green product knowledge can positively affect an individual’s purchasing decisions, which fully activates their GGF.

As a major reference group, family members’ pro-environmental concept and behavior have considerable demonstration effects, such that people become well-motivated to adopt green lifestyles (Moore et al., 2002; Lee, 2010) in order to acquire greater emotional attachment with their families. The pursuit of positive emotions can also provide individuals added pleasure and satisfaction (Lindenberg and Steg, 2007). Family identity driven by FAI can shape an individual’s HGF (Ivanova et al., 2019). If green products can also cut costs and avoid environmental damage to themselves and their family, individuals will have positive emotions and pleasurable experiences from purchasing green products (personal goals/achievements), further strengthening their hedonic motivations (Le et al., 2019).

Family is an important force in shaping environmental social norms (Bray et al., 2011). Ivanova et al. (2019) argue that family members have the strongest effect on forming environmental values during an individual’s childhood. Lee (2011) suggests that family members are an important force in the formation of individual environmental values and social norms. This effect is especially evident with the parents’ power of example, which has a behavioral demonstration effect on children, thus accelerating the formation of green social norms and their internalization into personal norms (Schwartz and Howard, 1981). Correspondingly, individuals consciously take social norms as common behavioral standards, generating NGF (Hafner et al., 2019). Hence, we posit the following hypothesis:

H2: *FAI positively affects consumers’ GGF (H2a), HGF (H2b), and NGF (H2c).*

#### Peer Influence and Goal Frames

Peer influence refers to how individual-specific behavior is affected by peers with similar cultural backgrounds, social customs, and values (Bristol and Mangleburg, 2005; Xu et al., 2017). Interpersonal interactions from opinion leaders and specialty people among friends may considerably influence individual cognitive activities (Tsarenko et al., 2013). Opinion leaders have high social status and authority and may play “active roles” on social occasions. When people are open-minded, they can accept new consumptive ideas and share recycling information that influences others (Xu et al., 2017). Following the GPB of key opinion leaders can strengthen the sense of belonging to specific groups, such as environmentalists, achieve gain and hedonic goals. This suggests that consumers’ purchasing motivations are not always driven by hedonic or utilitarian value but also by the need for identity salience and compliance with social norms (Andorfer and Liebe, 2013). Tsarenko et al. (2013) argue that peer approval to the green agenda can make individuals conform to the group norm and accept publicly identified environmental standards. In addition, Ivanova et al. (2019) suggest that friends, especially well-known persons, can profoundly influence consumers’ purchasing decisions. Therefore, we propose the following hypotheses:

H3: *PEI positively affects consumers’ GGF (H3a), HGF (H3b), and NGF (H3c).*

#### Goal Frames and Green Purchasing Behavior

This study defines GPB as doing environmentally friendly purchases that recycle or conserve resources or benefit the environment (Gonçalves et al., 2016; Mishal et al., 2017; Chen et al., 2020; Yang and Zhang, 2020). Previous studies have found a positive relationship between goal frames and pro-environmental behavior (Tang et al., 2020). Wang Y. et al. (2019) argue that when consumers realize green products can meet egoistic demands, they usually purchase them. Utilitarian appeal becomes the primary motivation for consumers to purchase green products (Chen, 2013). Wang Y. et al. (2019) also concluded that the top three motivations for consumers to purchase green products are: safety and health, reliable quality, and cost savings. Chen (2013) confirmed that green perceived value accomplishes customer loyalty to green products by gaining customer satisfaction and trust, thus realizing repeat purchasing behavior.

Emotional appeal is also an important factor affecting pro-environmental behavior (Meneses, 2010; Gonçalves et al., 2016). Previous studies have explored the impact of mood and affective experiences on environmental behavior (e.g., Lindenberg and Steg, 2007). Wang et al. (2017) found that positive emotional appeals effectively promote consumers’ willingness to purchase green products. Positive emotions, such as admiration and pride, are said to be important psychological motivations that can drive consumers toward green products (Tang et al., 2020).

Personal norms can be activated when buyers become aware of their environmental responsibilities or realize adverse consequences of their behavior (Schwartz and Howard, 1981; Zhang et al., 2013; Ge et al., 2020). Hafner et al. (2019) concluded that normative information is highly persuasive in driving an individual’s pro-environmental behavior. The greater the perceived environmental responsibility, the stronger the consumers’ environmental concern (Yang and Zhang, 2020). Similarly, Yarimoglu and Binboga (2019) argue that environmental concerns positively influence consumers’ pro-environmental behavior. Based on these arguments, the following hypothesis is proposed:

H4: *GGF (H4a), HGF (H4b), and NGF (H4c) positively affect consumers’ GPB.*

### Moderated Effects of Sensitivity to Climate Change

Sensitivity to climate change consists of two parts. First is the consumers’ concern for potential problems and challenges resulting from climate change, and second is their intention to realize harmonious coexistence between man and nature (Cheng and Wu, 2015). Cheng and Wu (2015) argued that individuals with high environmental sensitivity pay much greater attention to the natural environment. According to the five-factor model of personality (Costa and McCrae, 1992), individuals with high SCC tend to experience tension and anxiety toward climate change (Wang L. et al., 2019). In contrast, individuals with low SCC are generally unruffled, detached, and apathetic regarding climate change issues. For the high-SCC subgroup, their environmental concern and perceived seriousness of environmental problems (Wang L. et al., 2019; Xu et al., 2020) allow social influence to activate their three goal frames more easily and adopt more GPBs (Tang et al., 2020). This high SCC subgroup actively embraces GPB to mitigate climate change damages (Cheng and Wu, 2015) and assume environmental responsibilities (Hosta and Zabkar, 2021). Conversely, consumers in the low SCC subgroup are generally immune to social influence (Yang and Zhang, 2020) and often averse to external incentives concerning environmental issues. They may even show excessively strong perceived effectiveness of environmental behavior resulting in overconfidence (Yang and Zhang, 2020), which then weakens the activation ability of SI on three goal frames and reduces their acceptance to GPB. Based on these arguments, the following hypothesis is proposed:

H5: *Compared with the low SCC subgroup, consumers in the high SCC subgroup are more affected by the influence of ME, FAI, and PEI, which more easily activates their three goal frames and makes them more receptive to GPB.*

## Research Methodology

### Samples and Procedure

**FIGURE 2:**
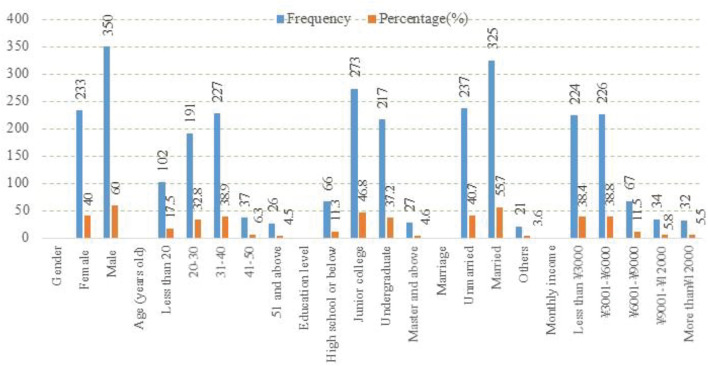
Respondent demographics (*N* = 583).

Urban residents from cities located in the Yangtze River Delta were surveyed and analyzed in this study. The Yangtze River Delta is one of China’s most economically developed areas, thus ensuring sample representativeness and generalizability (Yang and Zhang, 2020). The formal survey was conducted in February 2021 using online questionnaires,^1^ and little paper questionnaires were obtained from friends around the authors. Four filtering criteria were employed to filter out invalid samples: social desirability bias, cognitive bias for green consumption, incomplete survey responses, and respondents not from the Yangtze River Delta. From 731 questionnaires distributed, 583 questionnaires were valid, resulting in an effective rate of 79.75%. The demographic profile of respondents is summarized in Figure 2.

### Questionnaire Design

The components and metrics used in the questionnaire design were based on previous research. The elements for ME were adopted from Lee (2011), while those for family and PEIs were from Ivanova et al. (2019). Goal frames measuring gain, hedonic, and normative goals were adopted from Tang et al. (2020), the measure for GPB was borrowed from Yang and Zhang (2020), and four items for SCC was derived from Cheng and Wu (2015); Yang and Zhang (2020), and Xu et al. (2020).

**FIGURE 3:**
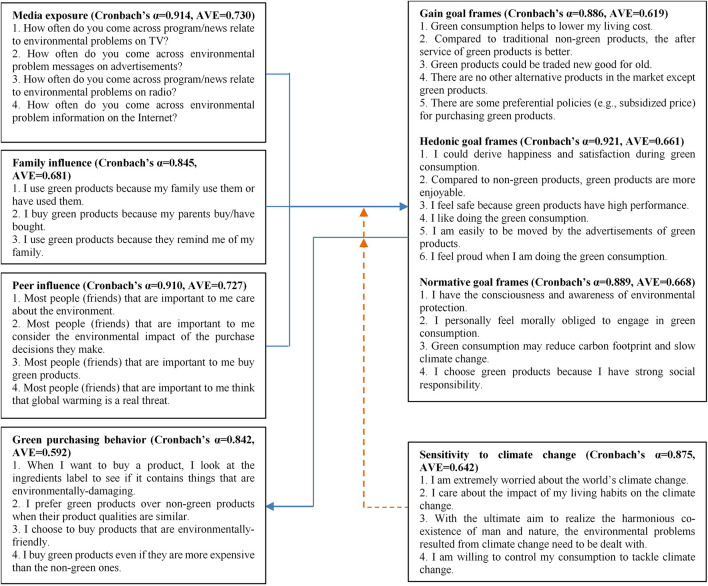
Constructs and items.

The questionnaire contains three parts. Part 1 provided the survey introduction, stating that the survey was to be used for academic and research purposes. It also defined and explained the meaning of green products. Part 2 contained the items for corresponding constructs, while Part 3 contained questions on the respondent’s personal information. To detect potential social desirability bias, we asked the respondents to answer the following questions: “What green products did you purchase in the last 3 months?” and “How many did you pay for green products in the last 3 months?” (Yang and Zhang, 2020). The technique of translation and back translation was used to ensure equivalence across languages. We sent the scales to five professors or doctors whose research interests included consumer behavior. A number of discussions, checks, and modifications were conducted to fit the green consumption context and study object. A 7-point Likert scale was used to quantify the responses, from (1) “*strongly disagree or not at all*” to (7) “*strongly agree or always.*” A pilot was conducted in December 2020, and the final scales were formed using exploratory factor analysis and reliability analysis. The constructs and final items are shown in Figure 3.

### Common Method Bias

Since all the variables were obtained using the same method, the results could potentially have common method bias (CMB), generating artificially inflated relationships (Podsakoff et al., 2003). Several processing control strategies were used to mitigate CMB, including maintaining respondent anonymity, hiding research purposes, and counterbalancing item orders (Le et al., 2019). We also conducted Harman’s test to analyze potential CMB. The results show that the first factor explained about 40% of the total variance, suggesting CMB was not a serious threat.

## Analysis and Results

### Measurement Model Analysis

The data analysis followed a two-step approach proposed by Anderson and Gerbing (1988). The first step performed confirmative factor analysis (CFA) to verify the validity and reliability of all constructs using AMOS 23 *via* the maximum likelihood estimate. The main results of the model analysis are as follows: *χ*^2^/df (1438.139/499) = 2.882, RMSEA = 0.057, CFI = 0.939, GFI = 0.865, TLI = 0.931, IFI = 0.939, NFI = 0.910, SRMR = 0.048. These values indicate that the goodness-of-fit for the measurement model was nearly acceptable and that the data fit the model well, given the common cutoff criteria (χ^2^/df < 5, RMSEA < 0.1, CFI > 0.9, GFI > 0.9, TLI > 0.9, IFI > 0.9, NFI > 0.9, SRMR < 0.08).

In Figure 3, the calculated Cronbach’s α coefficient for each scale was above 0.7, indicating good internal consistency (Hair et al., 2010). Convergent validity was tested using composite reliability (CR), standardized factor loading (derived from CFA), and average variance extracted (AVE), and the summary of results is presented in Table 1. The CR value for each scale was above 0.7 (Bagozzi and Yi, 1988; Hair et al., 2010), the AVE values for all constructs was greater than 0.5 (Fornell and Larcker, 1981), and the standardized factor loading for all items ranged from 0.592 to 0.911 (Hair et al., 2010). These calculated scores meet the recommended criteria. Also, the square root of AVE for each construct was found to be larger than the correlation between factors, indicating the adequacy of discriminant validity (Fornell and Larcker, 1981; Hair et al., 2010).

**TABLE 1:** Descriptive statistics, discriminate validity, and correlations among variables.

Variables	ME	FAI	PEI	GGF	HGF	NGF	GPB	SCC
ME	**0.854**							
FAI	0.514**	**0.825**						
PEI	0.687**	0.574**	**0.853**					
GGF	0.527**	0.582**	0.633**	**0.787**				
HGF	0.699**	0.570**	0.730**	0.718**	**0.813**			
NGF	0.621**	0.458**	0.678**	0.589**	0.710**	**0.817**		
GPB	0.535**	0.495**	0.635**	0.554**	0.662**	0.659**	**0.769**	
SCC	0.496**	0.308**	0.434**	0.254**	0.459**	0.481**	0.459**	**0.801**
CR	0.915	0.863	0.914	0.890	0.921	0.889	0.851	0.877
Mean	5.410	4.986	5.274	4.810	5.328	5.570	5.247	5.892
SD	1.119	1.199	1.112	1.170	1.047	0.968	1.080	0.965

The arithmetic square roots of average variance extracted (AVE) are on the diagonal in parentheses. *p < 0.05, **p < 0.01.

### Structural Model Analysis

The path coefficients among the latent variables were evaluated using AMOS 23. The fit statistics of the structural model achieved the recommended criteria (*χ*^2^/df = 3.453, GFI = 0.862, CFI = 0.931, TLI = 0.923, IFI = 0.931, NFI = 0.906, RMSEA = 0.065, SRMR = 0.057). The results of the path analysis show that ME had significant positive influences on HGF and NGF, but not significant for GGF (β_*ME*→*GGF*_ = 0.052, *p* > 0.1; β_*ME*→*HGF*_ = 0.318, *p* < 0.001; β_*ME*→*NGF*_ = 0.282, *p* < 0.001). Thus, H1b and H1c are confimed, while H1a is rejected. FAI was also found to have a significant positive effect on GGF and HGF (β_*FAI*→*GGF*_ = 0.252, *p* < 0.001; β_*FAI*→*HGF*_ = 0.163, *p* < 0.001), but not significant for NGF (β_*FAI*→*NGF*_ = 0.065, *p* > 0.1). This means hypotheses H2a and H2b are confirmed, while H2c is rejected. PEI was found to positively influence GGF, HGF, and NGF (β_*PEI*→*GGF*_ = 0.540, *p* < 0.001; β_*PEI*→*HGF*_ = 0.483, *p* < 0.001; β_*PEI*→*NGF*_ = 0.519, *p* < 0.001), confirming hypotheses H3a, H3b, and H3c. The results also showed HGF and NGF have significant positive influence on GPB (β_*HGF*→*GPB*_ = 0.288, *p* < 0.001; β_*NGF*→*GPB*_ = 0.428, *p* < 0.001) while GGF have an insignificant influence on GPB (β_*GGF*→*GPB*_ = −0.015, *p* > 0.1). This means hypotheses H3b and H3c are confirmed, while but H3a is rejected.

### Potential Mediated Effects

After examining the relationships among the various constructs, potential mediated paths were then investigated. While the Causal Steps approach proposed by Baron and Kenny (1986) has been widely used in previous research, simulation studies have suggested bootstrapping as a more powerful estimation method to examine mediation effects (e.g., Preacher and Hayes, 2004; MacKinnon, 2008; Zhao et al., 2010). We used bootstrapping to test the product coefficients (MacKinnon, 2008; Zhao et al., 2010), and the summary of findings is presented in Table 2. Using bias-corrected and percentile bootstrapping techniques, the confidence interval (CI) for total indirect effects (Holbert and Stephenson, 2003) of FAI on GPB included zero while the total indirect effects of ME and PEI excluded zero. Moreover, the three direct paths of ME, FAI, and PI on GPB were not significant.

**TABLE 2:** The results of total mediated effects with bootstrapping approach.

Hypotheses		Point estimation	Product of coefficients		Bootstrap (5,000 bootstrap samples)			
				
					Bias-corrected		Percentile	
			
					95% CI		95% CI	
			
			SE	Z	Lower	Upper	Lower	Upper
					Total effects			
SI→GF→GPB	ME	0.124	0.075	1.653	−0.028	0.266	−0.027	0.267
	FAI	0.111	0.057	1.947	0.009	0.239	0.003	0.234
	PEI	0.438	0.086	5.093	0.285	0.623	0.280	0.616
					Indirect effects			
SI→GF→GPB	ME	0.177	0.055	3.218	0.087	0.311	0.074	0.287
	FAI	0.052	0.035	1.486	−0.006	0.135	−0.011	0.124
	PEI	0.293	0.072	4.069	0.176	0.478	0.156	0.446
					Direct effects			
SI→GF→GPB	ME	−0.053	0.069	−0.768	−0.191	0.078	−0.181	0.084
	FAI	0.059	0.045	1.311	−0.022	0.155	−0.023	0.153
	PEI	0.145	0.089	1.629	−0.023	0.329	−0.013	0.341

We estimated the CIs of specific indirect effects using PRODCLIN2 program developed by Mackinnon et al. (2007). The main results are as follows: ME→GGF→GPB (−0.0055, 0.0047, include 0), ME→HGF→GPB (0.0355, 0.1270, exclude 0), and ME→NGF→GPB (0.0557, 0.1559, exclude 0); FAI→GGF→GPB (−0.0212, 0.0174, include 0), FAI→HGF→GPB (0.0137, 0.0615, exclude 0), and FAI→NGF→GPB (−0.0053, 0.0504, include 0); PEI→GGF→GPB (−0.0473, 0.0445, include 0), PEI→HGF→GPB (0.0519, 0.1935, exclude 0), and PEI→NGF→GPB (0.1095, 0.2748, exclude 0). The results, presented in Table 3, suggest considerable differences in the effect size and significance of specific indirect effects.

**TABLE 3:** Results of specific indirect effects.

Variable	Mediator = GGF			Mediator = HGF			Mediator = NGF		
			
	Effect	Lower	Upper	Effect	Lower	Upper	Effect	Lower	Upper
ME	0.0006	−0.0055	0.0047	0.0769	0.0355	0.1270	0.1010	0.0557	0.1559
FAI	0.0027	−0.0212	0.0174	0.0343	0.0137	0.0615	0.0203	−0.0053	0.0504
PEI	0.0065	−0.0473	0.0445	0.1153	0.0519	0.1935	0.1839	0.1095	0.2748

### Moderated Effects of Sensitivity to Climate Change

We used the test approach based on existing literature (e.g., Lin and Huang, 2012; Kautish et al., 2019) and split the samples into upper (last 73 percent) and lower (top 27 percent) subgroups, each containing about 27% of the total sample population (Kelley, 1939). The upper subgroup comprised 175 cases, while the lower subgroup was composed of 162 cases. To test group differences, we set all the paths equal among the constructs (Fully-constrained model) and compared the fully constrained and default model across upper and lower SCC (overall test). The results show that the Δχ^2^ varied significantly (Δ*χ*^2^ = 83.412, *p* < 0.001). As shown in Table 4, 15 constrained models were set for specific paths. The results suggest that the influence of FAI on HGF and NGF, PEI on NGF, and GGF, HGF, and NGF on GPB varied significantly for the upper and lower SCC groups. PEI can activate the normative goals in the lower SCC group more effectively than in the upper SCC group. In comparison, FAI has a stronger activation effect on the normative goals for the upper SCC group. The results also show that the two groups were not different in other paths. Therefore, H5 was partially confirmed.

**TABLE 4:** Moderated effects test.

	Lower SCC	Upper SCC	Model comparison		
			
	Standardized coefficients		Default model CMIN = 1666.935, DF = 774		
		
Paths	β_*Lower SCC*_	β_*Upper SCC*_	Restrained model DF = 775	ΔCMIN	Results
ME→GGF	0.133	0.060	1667.241	0.305	L = U
ME→HGF	0.241**	0.318***	1668.343	1.408	L = U
ME→NGF	0.223*	0.060	1668.868	1.933	L = U
FAI→GGF	0.243***	0.353***	1669.239	2.304	L = U
FAI→HGF	0.071*	0.287***	1675.380	8.445	L≠U
FAI→NGF	–0.112	0.248**	1683.946	17.011	L≠U
PEI→GGF	0.422***	0.359***	1669.293	2.358	L = U
PEI→HGF	0.517***	0.307***	1682.412	15.477	L = U
PEI→NGF	0.591***	0.288**	1683.186	16.251	L≠U
GGF→GPB	–0.049	0.193*	1675.832	8.897	L≠U
HGF→GPB	0.486***	–0.103	1679.212	12.277	L≠U
NGF→GPB	0.036	0.483***	1680.372	13.437	L≠U
ME→GPB	–0.062	0.048	1669.166	2.231	L = U
FAI→GPB	0.017	0.063	1667.280	0.345	L = U
PEI→GPB	0.303**	0.111	1668.596	1.661	L = U
Overall test			1750.348	83.412	L≠U

L, Lower SCC, U, Upper SCC. *p < 0.05, **p < 0.01, and ***p < 0.001.

## Discussion and Implications

### Findings and Discussion

This research is one of the few studies integrating Social Influence Theory with the Goal-framing Theory based on the S-O-R model. A number of interesting findings were observed.

First, this study proposed an integrated model to provide a robust framework for predicting consumers’ GPB. The results have provided deeper insights into identifying different effects of media, family, and peer on three goal frames and a new understanding of the impact of FAI. As hypothesized, family and PEI had significant positive effects on GGF. The results further support the original idea of the Diffusion of Innovations Theory in the green context, which posits that personal interactions are more persuasive than media influence. A possible explanation for this might be explained by the findings of Lee (2010), who stated that family and PEI could activate consumers’ egoistic appeals through the dissemination of environmental information and knowledge or personal demonstration. Consumers generally focus on the utilitarian value of green products when facing external incentives from environmental problems. If green products can provide protection from environmental crises (functional value) and deliver additional benefits (conditional value), GGFs can easily be generated (Chen, 2013). Oddly, the influence of media on GGFs was not significant, which could be caused by the respondents’ low trust in media advertising on green products (Chen et al., 2020).

Furthermore, our results also showed that media and PEI simultaneously had a significant positive effect on HGFs and NGFs, while FAI was only significant on HGFs. This finding is consistent with the findings of Lee (2011), who found that media and PEI affect consumers’ biospheric value. It can thus be concluded that consumers generate convergence motives and comply with social norms under group pressure to achieve self-categorization into the desired group (Hosta and Zabkar, 2021). Similar to the conclusions of Lee (2010), media, family, and peer effects caused consumers to have strong adverse opinions toward environmental problems that shape man-nature orientation (Jiang et al., 2020). This causes consumers to generate hedonic motives and tend to seek pleasure from pro-environmental behaviors.

Contrary to expectations, FAI did not significantly impact NGFs while significantly influenced GGFs. One possible explanation is that family members tend to show themselves when dealing with environmental problems and mainly communicate egoistic values with their relatives due to close family relationships. These findings reported here shed new light on expanding GPB from flexibly applying social groups, thus extending the current studies such as Lee (2011); Ivanova et al. (2019), and Suki and Suki (2019).

Second, the results provided a clearer picture of how consumers’ multiple motivations influence GPB and will further prove useful in expanding our understanding of the role of three goals (motivation) in guiding GPB. Similar to the conclusions of previous studies (e.g., Lindenberg and Steg, 2007; Tang et al., 2020), HGFs and NGFs significantly affect GPB. Surprisingly, GGFs activated by social influence did not exhibit a significant effect on GPB, extending studies, such as those by Tang et al. (2020). These results support the idea that HGFs and NGFs activated by social influence have predictable validity on consumers’ GPB, which was also reported by the Goal-framing Theory, proving that green social norms can effectively promote eco-friendly consumption (Schwartz and Howard, 1981; Zhang et al., 2013). Activating consumers’ environmental consciousness and ethics is highly effective in establishing a green consumption society (Kautish et al., 2019).

With respect to the hedonic goals, the outcome seems to be consistent with other research which found that if positive emotional experiences (e.g., pleasure, appreciation, and pride) can be generated from green consumption, consumers will actively develop GPBs (e.g., Wang et al., 2017; Tang et al., 2020). In addition, the unanticipated result regarding gain goals is similar to the findings of Lin and Huang (2012) but contrary to Wang et al. (2017). This outcome may be explained by the fact that reported in Lin and Huang’s (2012) investigation, 73% of the respondents could not accurately identify green products. Major obstacles that cause consumers to hold negative and skeptical attitudes toward green products include high prices, information asymmetry, poor availability, and greenwashing (Chen et al., 2020). Biswas and Roy (2015) also concluded that contextual factors, such as high prices, negative PEIs, and monotonous green product types, negatively influence perceived functional value (β = −0.27, *p* < 0.001), which can inhibit GPB and resulted in the motivation-behavior gap.

Third, this study exhibited the generation logic of GPB by demonstrating the potential mediation effects in the research model, thereby enriching the study of Lindenberg and Steg (2007) that goal frames were activated by stimuli, which can influence consumers’ GPB. Concretely, we found that goal frames have significant total mediation effects on the paths of ME and PEI on GPB but were not significant in mediating the relationship between FAI and GPB. The findings provide strong evidence that media and peers can effectively activate consumers’ HGFs and NGFs, to reach GPB. This conclusion supports prior studies which found that emotional (Wang et al., 2017) and normative appeals (Lindenberg and Steg, 2007), activated by social influence, can positively influence consumer attitude toward green products. However, our results did not find a significant mediation effect of GGFs. One possible reason is the lack of maturity of the current green consumption market, characterized by low innovation in green products, token environmental gestures (Chen et al., 2020), and fuzziness in the utility and satisfaction of green product use. Thus, positive gain goals fail to convert into actual green consumption, leading to a considerable gap between green motivation and behavior. Similarly, HGFs and NGFs significantly mediate the relationship between PEI and GPB. This supports previous research (Lee, 2010, 2011), which suggests peers are able to persuade others to be concerned about environmental affairs, follow green norms (Tsarenko et al., 2013), and care about their own feelings, emotions, and self-image in society (Wang L. et al., 2019).

Finally, the moderated effects of SCC were found to be partially significant as hypothesized. This new understanding should help extend our knowledge of consumer segmentation and shed new light on the difference in the motivation-behavior gap in lower and upper subgroups. For specific paths, the activation ability of FAI on HGFs and NGFs in the upper subgroup was significantly stronger than in the lower subgroup. A possible explanation is that consumers with high SCC, resulting from FAI, are more inclined to consider their family members’ fears and anxiety for environmental concerns, making them more ecologically conscious and environmentally responsible (Yang and Zhang, 2020; Hosta and Zabkar, 2021). However, one unanticipated finding was that the activation ability of media on the three goal frames had no significant difference between subgroups. Also, the activation ability of PEI on the NGFs in the lower SCC subgroup was significantly stronger than in the upper SCC subgroup. These results suggest that high SCC can cause excessive anxiety and fear (Janis and Feshbach, 1953). This can cause consumers to make irrational interpretations and infer that environmental crisis information reported from media and peer is deliberately exaggerated. Consumers would believe that the harm caused by environmental crises is less likely to happen (Yang and Zhang, 2020, 2021), alleviating themselves to bear environmental responsibilities. Significant differences were found between the upper and lower SCC subgroups in how the various goal frames convert into GPB. The influence of GGFs and NGFs on GPB in the lower SCC subgroup is significantly weaker than in the upper SCC subgroup. This suggests that the environmental crisis effectively activates consumers’ dual appeals, inducing them to purchase more green products to buffer against environmental problems and uncertainties (Xu et al., 2017). The appropriate perceived seriousness of environmental problems can make consumers embrace sustainable living and go green (Moon et al., 2019). Consequently, we can conclude that overcome lucky idea resulting from SCC, consider the dual appeals together, and provide guidance for each subgroup can contribute in several ways to expand GPB and narrow the motivation-behavior gap.

### Theoretical Implications

This research yielded the following theoretical implications. First, this study theoretically integrates three social factors and extends the social influence theory in the context of green consumption. As an acquired behavior, GPB results from the joint influence of various social forces. Social influence was partitioned into three dimensions (media, family, and peer) to comprehensively analyze the formation mechanism in consumer behavior. Second, this paper used three goal frames as mediators and systematically evaluated the complex psychological mechanism of green consumption. A three-dimensional goal motivation was generated that considers egoistic and altruistic appeals and comprehensively explored green consumption behavior to understand the root cause of the green motivation-behavior gap. Our findings suggest that consumers adopt GPBs due to multiple goal motivations. We also established a more robust research framework to detect potential causes of the motivation-behavior gap. Third, this paper makes a vital theoretical contribution by observing the significant moderating effect of SCC. Our findings provide a deeper insight into understanding the differences in GPB between the subgroups. This new understanding can help policymakers and marketers to improve predictions of expanding GPB with different psychological traits. Thus, this study enriches the literature on green consumption and provides greater understanding of how to segment customer groups and narrow the green motivation-behavior gap.

### Practical Implications

This work has a number of practical implications for decision-makers. Based on the study’s findings, policymakers need to pay attention to the differences in the activation ability of social influence on consumers’ goal frames. Given the major role of PEI in promoting GPB, policymakers should attach much importance to the guiding role of peers, account for dual appeals, and use positive emotional reinforcements, such as pride and admiration. Also, good market segmentation should be made, thereby precisely fitting consumers’ appeals for practical interests, strengthening environmental education, and helping consumers understand the functional, emotional, and social values of green products. Our results also show that the consumers’ normative motivations should be activated to instill long-term sustainable lifestyles.

The findings provide new insights on how to improve green marketing. Marketers should increase investments in green product innovation, follow the general law of the product life cycle, and continuously improve the output capacity of green products, overcoming the consumers’ perceived greenwashing and enhancing green functional value and brand trust (Chen et al., 2020). To end token environmental behavior, enterprises should integrate the driving effects of various social forces and focus on the mediation effects of hedonic and normative goals. Marketers may consider introducing green branding strategies, fully release the influencing power of ME and PEI, and assist consumers in identifying green products, providing green products the ability to meet consumers’ needs for positive emotions, and activating the shaping power of FAI through goal frames. Undoubtedly, symbolic value embodied in green branding can effectively persuade consumers to buy more green products and steadily promote GPBs by shaping social norms.

Finally, the findings of this research indicate that the effects of social influence on consumers with different environmental consciousness vary. Green practitioners need to flexibly use and optimize various social forces (e.g., media, peers) to more effectively activate consumers’ goal frames in promoting GPB. Likewise, a target market strategy could be adopted to make green products fit potential subgroups better and persuade them toward green products. To realize a tripartite win among enterprises, society, and consumers, green practitioners should create incentives that accelerate new product diffusion and adopt new strategies to guide consumers to adopt more GPBs.

## Limitations and Future Research Direction

There are several limitations in this study that require further investigation and improvement. First, due to negative interference from uncertainties, such as social desirability bias, measuring real GPB is highly complex and difficult. This paper has taken steps to obtain actual GPB data, but more improvements can be made in future research. For instance, researchers can consider identifying real green consumers by observing actual consumption scenarios. Second, our conclusions are based on data derived from Chinese urban residents. Further studies may consider western residents as the research object and compare the results with our conclusions.

## Data Availability Statement

The raw data supporting the conclusions of this article will be made available by the authors, without undue reservation.

## Ethics Statement

Ethical review and approval was not required for the study on human participants in accordance with the local legislation and institutional requirements. Written informed consent for participation was not required for this study in accordance with the national legislation and the institutional requirements.

## Author Contributions

XY: conceptualization, investigation, and formal analysis. XY and YT: methodology. YT and BL: validation. XY, YT, and BL: writing original draft and writing review. All authors have read and agreed to the published version of this article.

## Conflict of Interest

The authors declare that the research was conducted in the absence of any commercial or financial relationships that could be construed as a potential conflict of interest.

## Publisher’s Note

All claims expressed in this article are solely those of the authors and do not necessarily represent those of their affiliated organizations, or those of the publisher, the editors and the reviewers. Any product that may be evaluated in this article, or claim that may be made by its manufacturer, is not guaranteed or endorsed by the publisher.

## Footnotes

1
https://www.wenjuan.com/
